# Age-Friendly University Designation: Recognition, Synergy, and Unlimited Possibilities

**DOI:** 10.1093/geroni/igaf122.1936

**Published:** 2025-12-31

**Authors:** Pamela Elfenbein

**Affiliations:** University of North Georgia, Dahlonega, Georgia, United States

## Abstract

Immediately upon becoming recognized as an Age-Friendly University (2019) by the Age-Friendly University Global Network, the University of North Georgia Institute for Healthy Aging (IHA) was created. The IHA was tasked with a three-part mission: enhance the university’s academic gerontology programming, develop research partnerships and initiatives, and engage with community stakeholders to improve the quality of life for older adults in our region. With the recognition and synergy provided by our AFU designation the Institute for Healthy Aging has developed multiple programs and initiatives to meet each of our mission mandates, including increasing gerontology course offerings and in collaboration with the Georgia Division of Aging Services creating an enhanced associate’s degree to directly meet workforce needs, the development and facilitation of multiple gerontology focused grants and contracts, and, with community partners developed intergenerational programs and programs for older adults throughout our service region. This presentation will offer a timeline of the Institute for Healthy Aging’s academic, research, and community-based activities and programs in direct alignment with the Age-Friendly University Global Network Principles of an Age-Friendly University and the seven Domains of Age-Inclusive Practices.

